# The prevalence, risk factors, and prognostic value of venous thromboembolism in ovarian cancer patients receiving chemotherapy: a systematic review and meta-analysis

**DOI:** 10.1186/s12957-020-02101-5

**Published:** 2021-01-13

**Authors:** Lu Ye, Li Cai, Yonghui Fu, Debao Zhuang, Xiaoqing Hu, Youkun Jie

**Affiliations:** 1 Department of Pathology, Jiangxi Maternal and Child Health Hospital, Nanchang, 330006 Jiangxi China; 2 Department of Oncology, Jiangxi Maternal and Child Health Hospital, Nanchang, 330006 Jiangxi China; 3 Department of Psychiatry, Jiangxi Mental Hospital/Affiliated Mental Hospital of Nanchang University, Nanchang, 330029 Jiangxi China

**Keywords:** Venous thromboembolism, Ovarian cancer, Chemotherapy, Systematic review

## Abstract

**Background:**

Venous thromboembolism (VTE) in ovarian cancer (OC) patients has been widely investigated, but our knowledge on the role of VTE in OC patients receiving chemotherapy is limited. The aim of our study was to investigate the prevalence, risk factors, and prognostic value of chemotherapy-associated VTE in OC.

**Methods:**

Three databases (PubMed, Embase, and the Cochrane Library) were systematically searched from inception to October 14, 2020. The primary outcome was the prevalence of VTE in OC patients receiving chemotherapy. The risk factors and prognostic value of VTE were the secondary outcomes. The pooled prevalence of VTE was estimated using the generic inverse-variance method. The statistical heterogeneity was evaluated with Cochran’s *Q* test and *I*^2^ statistic. Funnel plot, Begg’s test, and Egger’s test were used to assess the potential publication bias in the meta-analysis.

**Results:**

A total of eleven observational studies with 4759 OC patients were included. The pooled prevalence of VTE was 9% (95% CI, 0.06–0.12) in OC patients receiving chemotherapy. The results of subgroup analysis and sensitivity analysis were basically consistent with the overall pooled estimate. Multiple significant risk factors associated with VTE were also identified including advanced age, D-dimer > 0.5 mg/mL, and tumor diameter > 10 cm. Only two included studies reported the prognostic value of VTE in OC patients receiving chemotherapy, but with inconsistent results. Funnel plot showed that there existed potential publication bias, which was further verified by statistical test, but the results of the trim-and-fill method showed the pooled estimate kept stable after adding two “missing” studies.

**Conclusions:**

This current study revealed that the pooled prevalence of chemotherapy-related VTE in OC was approximately 9% in OC patients. Risk factors for chemotherapy-related VTE were also identified which may contribute to targeting potentially preventative measures for VTE in OC.

**Supplementary Information:**

The online version contains supplementary material available at 10.1186/s12957-020-02101-5.

## Introduction

Venous thromboembolism (VTE), a known complication of malignancy, frequently develops in patients with ovarian cancer (OC) with a relatively high incidence [1–3]. Moreover, thrombotic events are associated with increased morbidity, mortality, and impaired quality of life in patients with OC [4, 5]. VTE may lead to increased hospital costs, which largely imposes additional burdens to the medical systems [5, 6]. Numerous risk factors are identified to be linked to VTE in OC, including older age, low differentiated grade, D-dimer greater than 788 μg/L, PT greater than 11.7 s, and CA125 greater than 760 U/mL [7, 8]. In general, cytoreductive surgery together with postoperative chemotherapy is well-established management for OC, especially advanced OC [9–11]. It is well-known that surgery is an important inducer to the development of VTE, but increasing studies suggest that there also exists a positive correlation between chemotherapy and VTE in patients with malignancies [12, 13]. Understandably, it seems to be very essential to clarify the prevalence, risk factors, and prognostic value of VTE in OC patients receiving chemotherapy, which may provide potential individualized guidance on the use of chemotherapy-associated VTE prophylaxis in OC.

Actually, accumulating evidence indicates that VTE is not rare in OC patients undergoing chemotherapy, but the prevalences are inconsistent across these published studies [14–17]. For instance, Salinaro and coworkers found that nearly 7.7% patients were diagnosed with VTE after receiving neoadjuvant chemotherapy for advanced OC [17]. Chavan et al. revealed that there was a 13.6% incidence of VTE in patients undergoing neoadjuvant chemotherapy for OC [15]. Furthermore, many studies found that some risk factors were deemed as significant predictors of chemotherapy-associated VTE prophylaxis in OC, including ascites, age 55 years and older, and tumor diameter greater than 10 cm [14, 16]. However, there were no studies to systematically summarize these identified risk factors and quantitatively explore the correlative dimension between these factors and chemotherapy-associated VTE in OC. Additionally, previous studies showed that VTE was an independent predictor of decreased survival outcomes in OC, but whether the same prognostic value of VTE also applies to OC patients undergoing chemotherapy is unclear.

Considering the above disputes and inconsistencies, we performed a systematic review and meta-analysis to comprehensively explore the prevalence, risk factors, and prognostic value of VTE in OC patients receiving chemotherapy, which may be conducive to provide potentially preventative measures for VTE.

## Methods

This current meta-analysis was conducted in accordance with the guideline of the Meta-analysis of Observational Studies in Epidemiology checklist [18] and the Cochrane Handbook.

### Search strategy and study selection

PubMed, Embase, and the Cochrane Library were systematically searched from the inception to October 14, 2020, using the search strategy including the terms for “chemotherapy,” “thromboembolism,” “ovarian cancer,” and their variants. The detailed search strategy was shown in Additional file 1. Furthermore, we also checked the references of included studies and important reviews for any potential inclusion. Meanwhile, we searched unpublished studies through searching gray literatures including ClinicalTrials.gov, the ISRCTN metaregister of controlled trials, and World Health Organization trials page. We only included observational studies which explored the prevalence, risk factors, and prognostic value of VTE in OC patients receiving chemotherapy. Studies published in non-English language or published in non-full text (including abstracts) were excluded. For the same studies with different follow-ups, we merely included the one with the bigger sample size and longer duration. Endnote software was used to screen eligible studies according to the pre-defined inclusion criteria. Two reviewers (*Lu Ye* and *Li Cai*) independently undertook search strategy and study selection, with inconsistence resolved by the chief reviewer (*Youkun Jie*).

### Data extraction

A standardized data extraction form was used to extract the following data: year of publication; country or countries of origin; the demographic characteristics of OC patients; clinicopathological stage of OC; operative details; chemotherapy protocol; sample size; definition of VTE; the prevalence, risk factors, and prognostic value for VTE; confounders on multivariate analysis; and study design. Only adjusted RRs (relative risks) or ORs (odds ratios) with 95% CIs (confidence intervals) on multivariate analysis were extracted.

### Methodological quality

The quality of included studies was evaluated using the Newcastle-Ottawa Scale (NOS) score, which can be used to assess the quality of observational studies [19]. The score system contains three dimensions which include selection criteria of participants, comparability, exposure, and outcome. Every dimension was scored 3 points, and the maximum score of NOS is up to 9 [19].

### Statistical analysis

The primary outcome was the prevalence of VTE in OC patients receiving chemotherapy. The risk factors and prognostic value of VTE were the secondary outcomes. The pooled prevalence of VTE was estimated using the generic inverse-variance method described by DerSimonian and Laird [20]. The correlative dimension of risk factors with chemotherapy-related VTE was summarized as odds ratios (ORs) with 95% confidence intervals (CIs). Only ORs with 95% CIs on multivariate analysis were used for pooled analyses. The prognostic role of chemotherapy-associated VTE on the overall survival (OS), disease-free survival (DFS), and relapse-free survival (RFS) in OV was assessed using hazard ratios (HRs) with 95% CIs. Only meta-analyses were performed when three or more included studies reported the same outcomes of interest; otherwise, qualitative systematic reviews for them were conducted. The statistical heterogeneity was evaluated with Cochran’s *Q* test and *I*^2^ statistic [21, 22]. Considering the substantial clinical heterogeneity within or between included studies, random-effect model was used for all the meta-analyses in the current study. To explore the potential sources of heterogeneity in the current meta-analysis, we conducted meta-regression analysis to investigate the roles of sample size, NOS score, and year of publication in statistical heterogeneity. Moreover, subgroup analyses based on OC stages, sample size, study quality, chemotherapy regimens, and others were performed. Also, we undertook influence analyses to clarify the influence of individual included studies on the overall pooled estimate through removing one study each time. Funnel plot, Begg’s test, and Egger’s test were used to assess the potential publication bias in the meta-analysis [23, 24]. If there existed significant publication bias, subsequent sensitivity analysis using the trim-and-fill method was used to find the possible “missing studies” and further explore the effect of “missing studies” on the pooled effect estimate [25, 26].

## Results

### Study selection

**Fig. 1:**
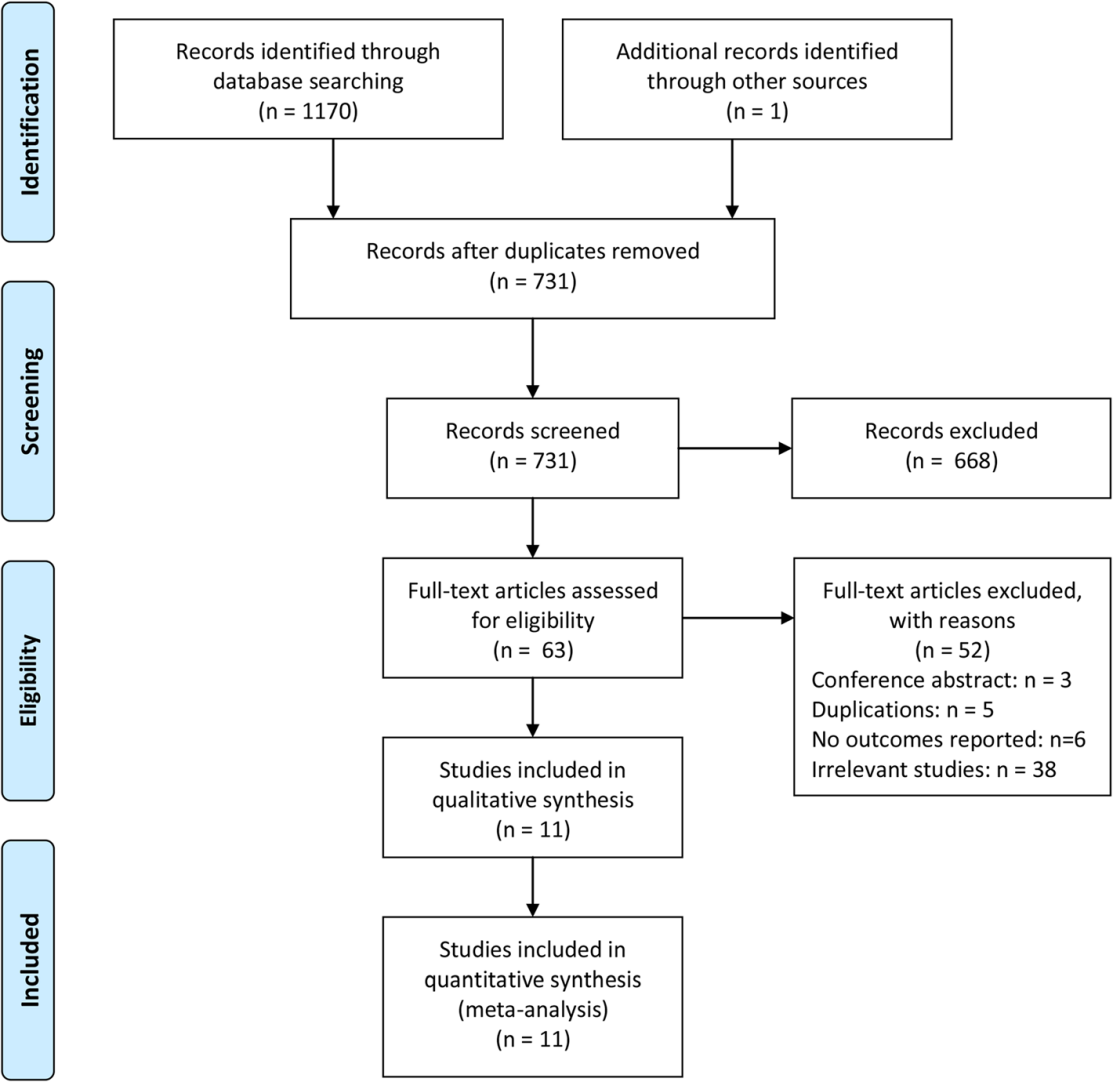
Flow diagram of the study selection in this meta-analysis

We performed an updated search on October 14, 2020. A total of 1170 items were obtained through initial literature search from three databases (PubMed, Embase, and the Cochrane Library). After removing duplicated items, the remaining 731 studies were further checked through screening relevant titles and abstracts. A total of 668 studies were identified to be ineligible to our pre-defined inclusion criteria. Subsequently, the remaining 63 studies underwent full-text checking, and 52 full-text articles met the exclusion criteria. Finally, 11 studies were included in the current meta-analysis (Fig. 1) [14–17, 27–33].

### Study characteristics

Of all the included studies, six were performed in Europe, three in the USA, and two in China. All the studies were published between 2008 and 2020 with sample size ranging from 18 to 2743. Also, the disease states of patients in included studies were different from each other. Of these, patients in four included studies had advanced OC, while patients in two included studies had recurrent OC. Furthermore, the chemotherapy regimens in all the included studies were also different from each other. As a whole, seven studies involved in adjuvant chemotherapy and six in neoadjuvant chemotherapy. The detailed baseline characteristics and chemotherapy regimens in included studies are shown in Tables 1 and 2. Additionally, we assessed the methodological quality of included studies using NOS score (Table 3). The whole NOS score of candidate studies ranged from 5 to 8 points. One study was scored 5 points, one 6 points, three 7 points, and six 8 points, which indicated the most of included studies were moderate to high quality.

**Table 1:** Characteristics of studies included in the meta-analysis

Author, year	Year of study	Country	Disease stage	Operation type	Chemotherapy type	No. of VTEs	No. of participants	VTE diagnostic criterion	Study design
Fotopoulou et al., 2008 [27]	1995–2002	Germany	Advanced ovarian cancer	Debulking surgery	Adjuvant chemotherapy	76	2743	National Cancer Institute Common Toxicity Criteria	Case-control
Fotopoulou et al., 2009 [14]	1999–2005	Germany	Recurrent ovarian cancer	Radical surgery	Adjuvant chemotherapy	37	525	National Cancer Institute Common Toxicity Criteria	Case-control
Mereu et al., 2009 [28]	1990–2004	Italy	Ovarian cancer	Radical surgery	Adjuvant chemotherapy	16	203	Self-definition	Cross-sectional study
Guardiola et al., 2010 [29]	2004–2007	France	Recurrent ovarian cancer	Debulking surgery	Neoadjuvant chemotherapy	3	18	National Cancer Institute Common Toxicity Criteria	Cross-sectional study
Saadeh et al., 2013 [30]	2006–2010	Ireland	Ovarian cancer	Radical surgery	Neoadjuvant chemotherapy or adjuvant chemotherapy	31	227	Self-definition	Case-control
Pant et al., 2014 [31]	2008–2011	The USA	Advanced ovarian cancer	Radical surgery	Adjuvant chemotherapy	16	128	Self-definition	Case-control
Chavan et al., 2017 [15]	2012–2015	China	Ovarian cancer	Radical surgery	Neoadjuvant chemotherapy or adjuvant chemotherapy	20	147	Self-definition	Retrospective cohort
Greco et al., 2017 [32]	2009–2014	The USA	Advanced ovarian cancer	Debulking surgery	Neoadjuvant chemotherapy	12	111	Self-definition	Retrospective cohort
Kuk et al., 2017 [33]	NA	Poland	Advanced ovarian cancer	Debulking surgery	Adjuvant chemotherapy	5	57	Self-definition	Case-control
Zhang et al., 2018 [16]	2014–2017	China	Ovarian cancer	Debulking surgery	Neoadjuvant chemotherapy	35	370	Self-definition	Case-control
Salinaro et al., 2020 [17]	2000–2013	The USA	Ovarian cancer	Debulking surgery	Neoadjuvant chemotherapy	16	230	Self-definition	Retrospective cohort

*VTE* venous thromboembolism

**Table 2:** Chemotherapy regimens of studies included in the meta-analysis

Author, year	Chemotherapy regimens
Fotopoulou et al., 2008 [27]	Platinum/paclitaxel-based chemotherapy.
Fotopoulou et al., 2009 [14]	Platinum-based first-line chemotherapy.
Mereu et al., 2009 [28]	The first-line chemotherapy schedules were as follows: cysplatinum (CDDP 50 mg/mq weekly), cysplatinum + other drugs (CDDP 50 mg/mq + cyclophosphamide 600 mg/mq T adriamycin 45 mg/mq every 3Y4 weeks), carboplatin (JM8 AUC6 every 3Y4 weeks), and carboplatin + other drugs (JM8 AUC6 + paclitaxel 175Y225 mg/mq every 3 weeks, JM8 AUC6 + epirubicin 120 mg/mq every 4 weeks, JM8 AUC5 + paclitaxel 175 mg/mq + topotecam 1 mg/mq per 3 days every 3 weeks).
Guardiola et al., 2010 [29]	Chemotherapy with platinum-containing regimens.
Saadeh et al., 2013 [30]	Adjuvant and neoadjuvant chemotherapy (details were unavailable).
Pant et al., 2014 [31]	Frontline adjuvant chemotherapy (details were unavailable).
Chavan et al., 2017 [15]	Adjuvant and neoadjuvant chemotherapy (details were unavailable).
Greco et al., 2017 [32]	Neoadjuvant chemotherapy (details were unavailable).
Kuk et al., 2017 [33]	The control group (26 patients) was treated with three to six cycles of standard first-line chemotherapy consisting of carboplatin (AUC 6 i.v.) and paclitaxel (175 mg/m^2^ i.v.) (CP). The study group (31 patients) was treated with CP chemotherapy with the addition of bevacizumab (7.5 mg/kg bw i.v.).
Zhang et al., 2018 [16]	Neoadjuvant chemotherapy with carboplatin paclitaxel was administered for 2 or 3 courses before cytoreductive surgery.
Salinaro et al., 2020 [17]	Neoadjuvant chemotherapy (details were unavailable).

**Table 3:** NOS scores of studies included in the meta-analysis

Author, year	Selection	Comparability	Outcome/exposure	Total score
Fotopoulou et al., 2008 [27]	****	**	**	8
Fotopoulou et al., 2009 [14]	****	**	**	8
Mereu et al., 2009 [28]	***	*	**	6
Guardiola et al., 2010 [29]	***	*	*	5
Saadeh et al., 2013 [30]	****	**	**	8
Pant et al., 2014 [31]	***	**	**	7
Chavan et al., 2017 [15]	****	**	**	8
Greco et al., 2017 [32]	****	**	**	8
Kuk et al., 2017 [33]	****	**	*	7
Zhang et al., 2018 [16]	****	**	**	8
Salinaro et al., 2020 [17]	****	**	*	7

### Prevalence of VTE

**Fig. 2:**
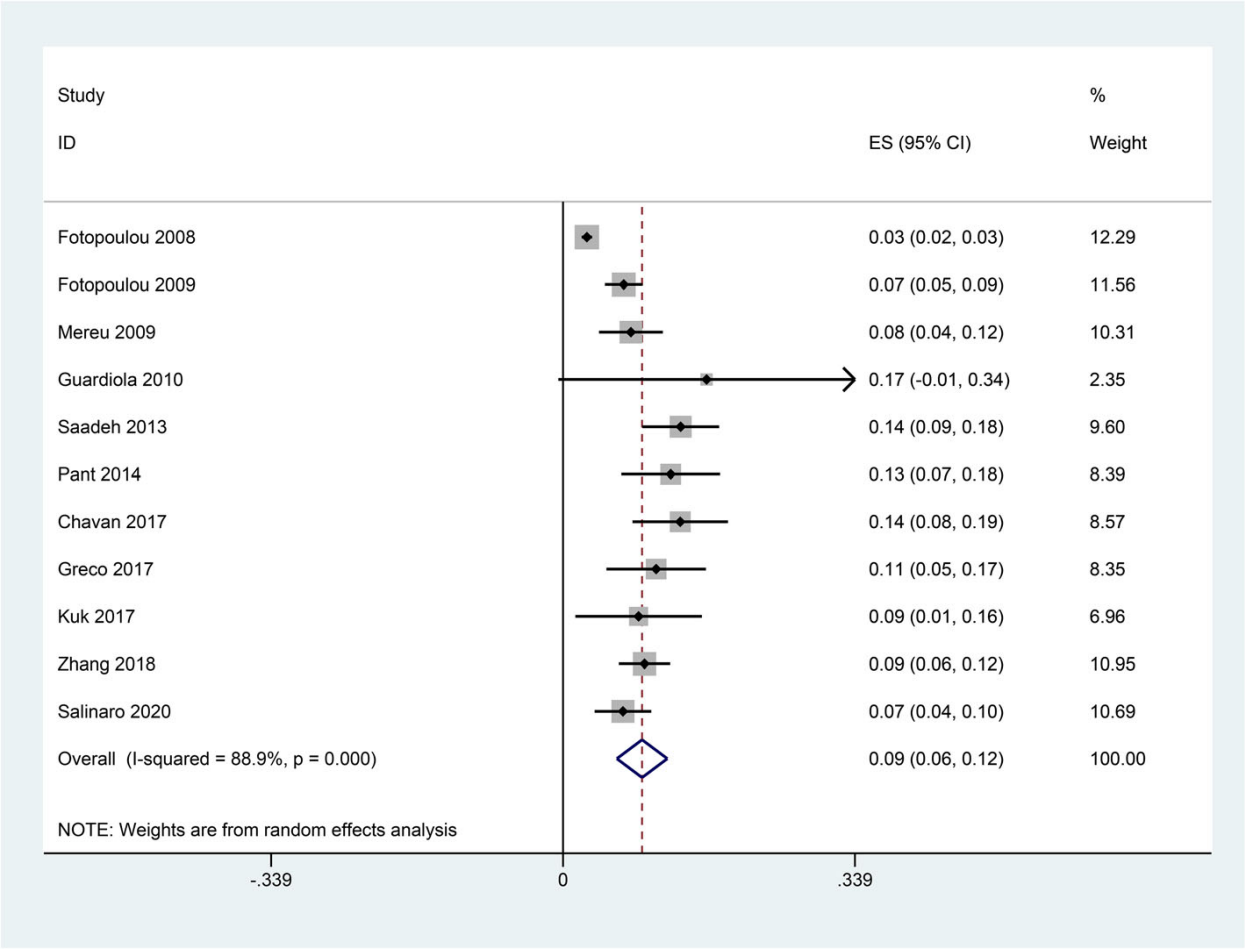
Forest plot for prevalence of venous thromboembolism (VTE) in ovarian cancer patients receiving chemotherapy

**Fig. 3:**
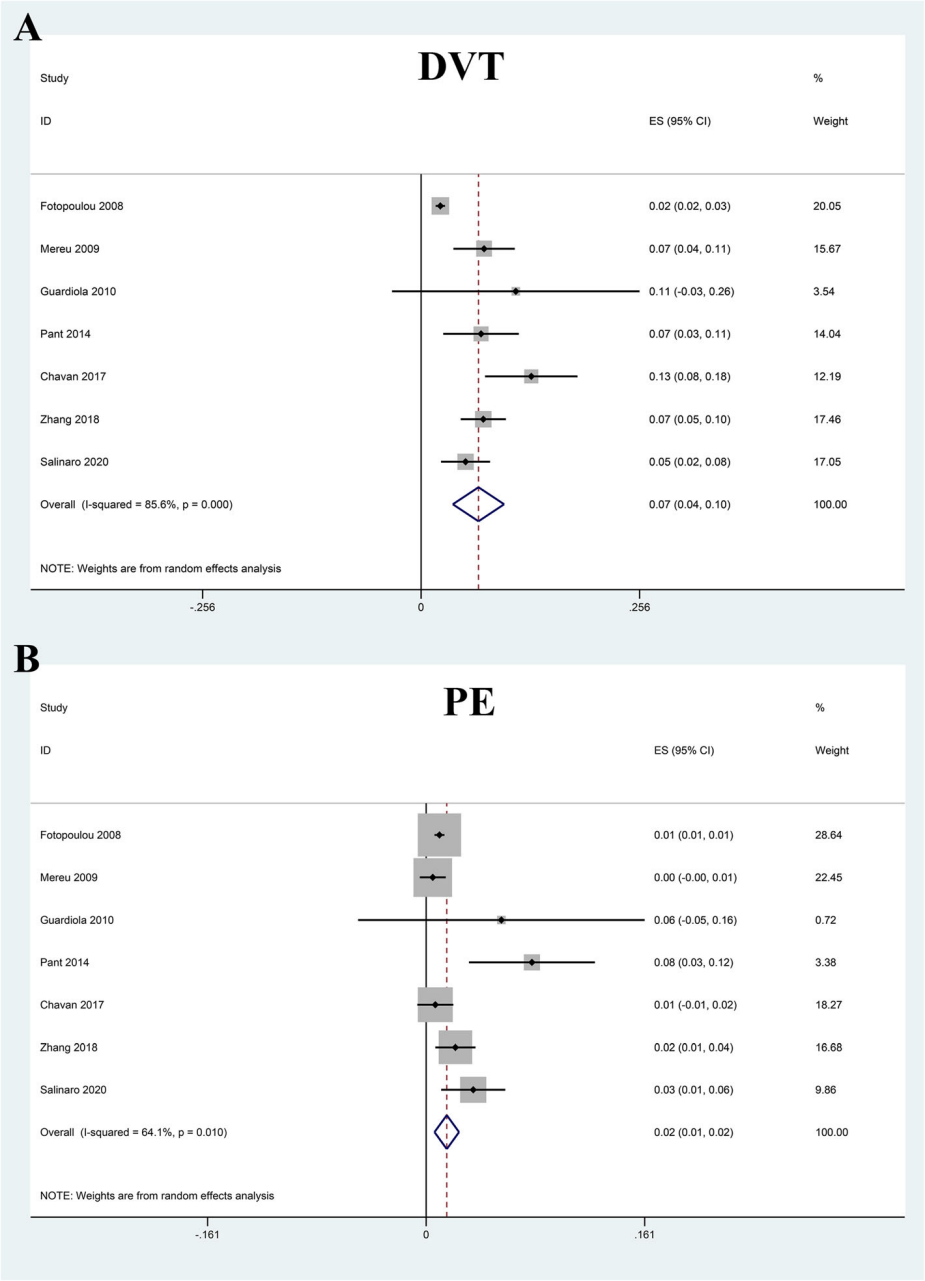
Forest plot for prevalence of deep venous thrombosis (DVT) and pulmonary embolism (PE) in ovarian cancer patients receiving chemotherapy

**Fig. 4:**
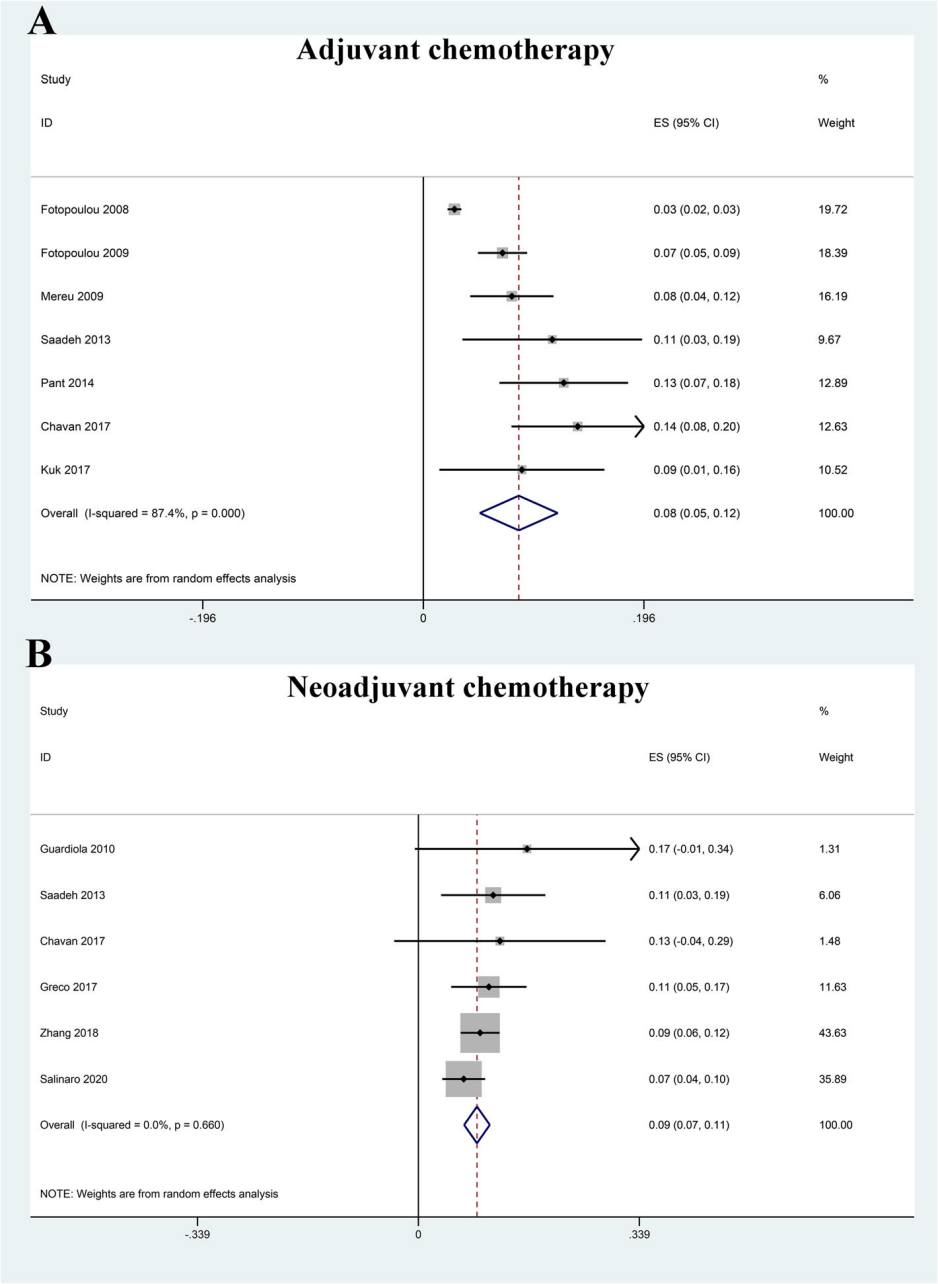
Forest plot for prevalence of venous thromboembolism (VTE) in ovarian cancer patients receiving adjuvant chemotherapy (**a**) and neoadjuvant chemotherapy (**b**)

**Fig. 5:**
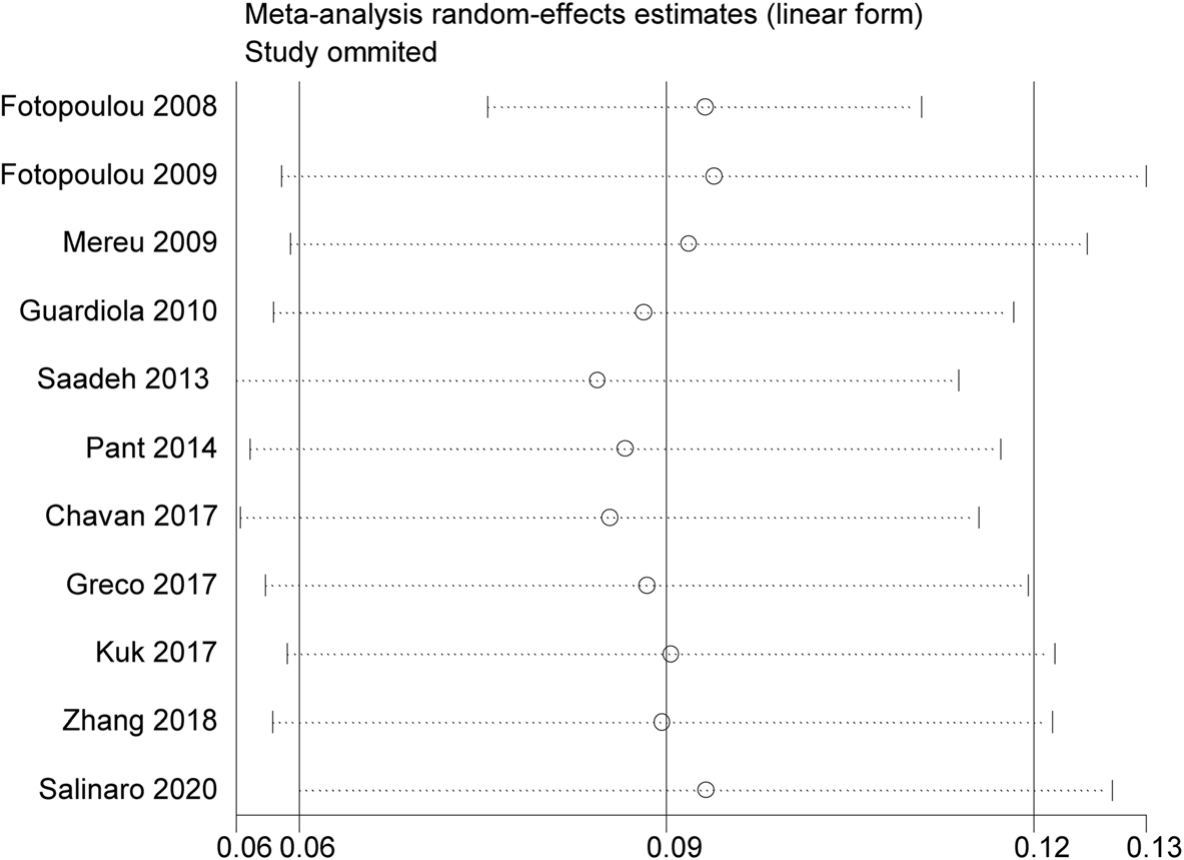
Sensitivity analyses for prevalence of venous thromboembolism (VTE) in ovarian cancer patients receiving chemotherapy

**Fig. 6:**
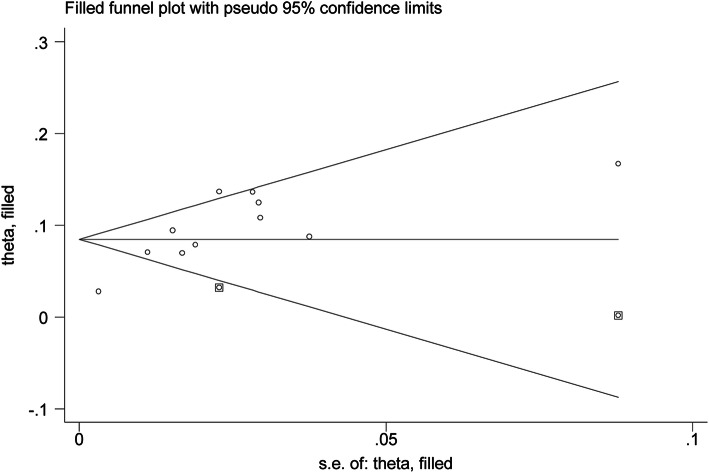
Adjusted funnel plot of chemotherapy-associated venous thromboembolism in ovarian cancer after adding two “missing” studies from the “trim-and-fill” analysis

All the included studies reported the number of VTE, and the pooled prevalence of VTE was 9% (0.06–0.12) with substantial heterogeneity (*I*^2^ = 88.9%) (Fig. 2). In general, VTE events are defined as deep vein thrombus (DVT) and pulmonary embolus (PE). Accordingly, we also explored the prevalence of DVT and PE in OC patients receiving chemotherapy. Our results indicated that the pooled prevalence of DVT and PE was 7% (0.04–0.10) and 2% (0.01–0.02), respectively (Fig. 3). Considering the significant heterogeneity across included studies, the meta-regression analyses based on sample size, publication time, and NOS score were used to explore the potential sources of statistical heterogeneity. The meta-regression analyses revealed that sample size (*p* = 0.002), but not publication time (*p* = 0.207) and NOS score (*p* = 0.817), may be the potential source of statistical heterogeneity. We also performed subgroup analyses based on different stratified standards including publication time, region, disease stages, operation type, sample size, VTE diagnostic criterion, study design, and NOS score. The pooled estimates of VTE in subgroups were basically consistent with the overall pooled effect except in those in publication time ≤ 2009 (0.06, 0.02–0.09), sample size > 500 (0.05, 0.01–0.09), and Common Toxicity Criteria (0.05, 0.01–0.10) (Table 4). The pooled prevalences of VTE in patients receiving adjuvant and neoadjuvant chemotherapy were 8% (0.05–0.12) and 9% (0.07–0.11), respectively (Fig. 4). The results of influence analysis kept stable with ORs with CIs ranging from 0.086 (0.057–0.115) to 0.096 (0.061–0.13) (Fig. 5). We further evaluated whether there existed potential publication bias in the current meta-analysis. The funnel plot seemed to be asymmetrical, which was further verified by statistic tests (Begg’s test: *p* = 0.755; Egger’s test: *p* = 0.003; Fig. 6). Considering the significant publication bias, sensitivity analysis using the trim-and-fill method was used to explore the effect of “missing studies” on the pooled prevalence of VTE. The results showed that two studies were “missing” in the current meta-analysis. Interestingly, the new pooled estimate (0.085, 0.058–0.111) was basically consistent with the previous one (Fig. 6).

**Table 4:** Subgroup analysis for the prevalence of venous thromboembolism in ovarian cancer patients receiving chemotherapy

Subgroups	No. of studies	Pooled rate with 95% CI	*I*^2^ (%)
The overall pooled result	11	0.09 (0.06, 0.12)	88.9
Publication time			
≤ 2009	3	0.06 (0.02, 0.09)	89.9
> 2010	8	0.10 (0.08, 0.12)	22.8
Region			
Asia	2	0.11 (0.07–0.15)	40
Europe	6	0.08 (0.04–0.12)	88.9
The USA	3	0.09 (0.06–0.13)	38.9
Disease status			
Ovarian cancer	5	0.10 (0.07–0.12)	52.7
Advanced ovarian cancer	4	0.08 (0.02–0.14)	85.4
Recurrent ovarian cancer	2	0.08 (0.03–0.13)	15.3
Operation type			
Debulking surgery	6	0.08 (0.04–0.12)	85.7
Radical surgery	5	0.10 (0.07–0.13)	65.7
Sample size			
≤ 500	9	0.10 (0.08–0.12)	22.2
> 500	2	0.05 (0.01–0.09)	92.6
VTE diagnostic criterion			
Self-definition	8	0.10 (0.08–0.12)	27.6
CTC	3	0.05 (0.01–0.10)	87.5
Study design			
Case-control	6	0.09 (0.05–0.13)	91.9
Cross-sectional study	2	0.08 (0.05–0.12)	0
Retrospective cohort	3	0.10 (0.06–0.14)	55.6
NOS score			
≤ 6	2	0.08 (0.05–0.12)	0
> 6	9	0.09 (0.06–0.12)	90.4

*CI* confidence interval, *CTC* Common Toxicity Criteria

### Risk factors for VTE in OV patients receiving chemotherapy

Only five studies reported multivariate or adjusted risk factors associated with VTE in OC patients receiving chemotherapy [14–16, 27, 28]. Owing to wide variations across these identified risk factors, we did not undertake meta-analyses for these variables quantitatively. We merely perform systematic reviews for them qualitatively (Table 5). Fotopoulou and colleagues found that age (35–81 years), BMI < 30 kg/m^2^, and ascites were significant risk factors for VTE [27]. Mereu et al. revealed that BMI and mono-chemotherapy were significant predictors for VTE [28]. Zhang and coworkers investigated that age > 55 years, PLT > 300 109/L, D-dimer > 0.5 mg/mL, and tumor diameter > 10 cm were significant risk factors for VTE. Of these, D-dimer > 0.5 mg/mL was the strongest predictor for VTE (OR 17.317; 95% CI, 3.485–86.057) [16].

**Table 5:** Risk factors associated with VTE on multivariate model in ovarian cancer patients receiving chemotherapy

Study	Risk factors on multivariate model
Fotopoulou et al. [27]	Age (35–81 years): HR 1.4, 95% CI 1.1–1.8; BMI < 30 kg/m^2^: HR 3.2, 95% CI 2.0–5.2; FIGO IIIc or IV: HR 1.0, 95% CI 0.6–1.7; chemotherapy: HR 0.2, 95% CI 0.1–0.7; ascites: HR 1.5, 95% CI 0.9–2.3; para-aortic lymphadenectomy: HR 0.5, 95% CI 0.3–1.0; pelvic lymphadenectomy: HR 1.1, 95% CI 0.6–2.0
Fotopoulou et al. [14]	Age < 60 years: HR 1.26, 95% CI 0.58–2.71; BMI < 30 kg/m^2^: HR 1.07, 95% CI 0.49–2.36; ECOG: HR 0.89, 95% CI 0.42–1.87; platinum-sensitive: HR 1.16, 95% CI 0.56–2.4; serous-papillary: HR 0.64, 95% CI 0.29–1.38; ascites: HR 2.2, 95% CI 1.02–4.72; surgery: HR 0.58, 95% CI 0.24–1.36; new relapse: HR 0.64, 95% CI 0.26–1.53
Mereu et al. [28]	Age: HR 1.27, 95% CI 0.94–1.70; BMI: HR 1.62, 95% CI 1.08–2.42; FIGO stage: HR 7.14, 95% CI 0.81–62.89; histologic diagnosis: HR 0.20, 95% CI 0.05–0.78; mono-chemotherapy: HR 4.97, 95% CI 1.50–16.49; risk category: HR 2.81, 95% CI 0.88–8.96
Chavan et al. [15]	Neoadjuvant chemotherapy: RR 0.90, 95% CI 0.23–3.56; menopausal: RR 3.2, 95% CI 0.95–10.09
Zhang et al. [16]	Age > 55 years: OR 13.110, 95% CI 2.451–70.133; PLT > 300 109/L: OR 3.987, 95% CI 1.085–14.657; D-dimer > 0.5 mg/mL: OR 17.317, 95% CI 3.485–86.057; tumor diameter > 10 cm: OR 4.93, 95% CI 1.364–17.819

### Prognostic value of VTE in OC patients receiving chemotherapy

Only two included studies reported the prognostic value of chemotherapy-associated VTE in OC patients [14, 27]. Fotopoulou et al. found that 1-year mortality in the VTE group was significantly higher than that in the non-VTE group [27]. Multivariate analysis indicated that neither PE nor DVT was associated with a higher OC recurrence [27]. Furthermore, another study revealed that VTE was not an independent prognostic factor for progression-free survival in OC patients receiving platinum-based first-line chemotherapy [14].

## Discussion

The current meta-analysis based on 11 observational studies indicated that VTE was a relatively common complication in OC patients receiving chemotherapy. The systematic review for risk factors found that some risk factors were potential predictors for VTE including advanced age, D-dimer > 0.5 mg/mL, and tumor diameter > 10 cm.

Our findings revealed that the pooled prevalence of chemotherapy-related VTE in OC was approximately 9% in OC patients with substantial heterogeneity (88.9%). Considering that significant heterogeneity may impair the evidence quality of the pooled estimate, the meta-regressions based on publication time, sample size, and NOS score were performed to explore the potential sources of statistical heterogeneity. We found that sample size may be an important reason responsible for significant statistical heterogeneity. Consistently, subgroup analysis based on sample size showed that the pooled prevalence of included studies with sample size > 500 was lower than that in sample size ≤ 500. We also performed sensitivity analysis to explore the robustness of the overall pooled estimate. The pooled effects of sensitivity analysis remained slight fluctuation, which suggested that the current pooled estimate was robust and credible. To clarify the prevalence of VTE in different clinical settings, we performed subgroup analysis based on publication time, region, disease stages, operation type, VTE diagnostic criterion, study design, and NOS score. We found that the prevalences were basically consistent with the overall pooled effect in subgroups based on region, disease stages, operation type, study design, and NOS score. The prevalence of VTE in the sub-populations with publication time ≤ 2009 was relatively lower than that in publication time > 2010. Understandably, the proportion of OC increased significantly in recent years [34, 35]. Machida et al. found that clear cell carcinoma has increased significantly between 2002 and 2010 which accounted for nearly 30% of epithelial OC in Japan [34]. Althubiti and coworkers showed that the incidence of OC increased around 4-fold in Saudi Arabia between 1990 and 2016 [35]. Interestingly, increasing evidence investigated that advanced age was a significant risk factor for VTE [36, 37]. Therefore, a possible interpretation is that the prevalence difference of VTE between publication time ≤ 2009 and > 2010 may be attributed to an aging population in recent years.

We also explored the potential risk factors associated with VTE in OC patients receiving chemotherapy. Some significant risk factors were identified to be significant predictors for chemotherapy-associated VTE including advanced age, ascites, D-dimer > 0.5 mg/mL, and tumor diameter > 10 cm. Actually, mountains of studies found that advanced age was a significant predictor for VTE in patients. Stämpfli and colleagues found that endothelial dysfunction was a critical inducer to VTE and aging was associated with endothelial dysfunction [38]. Further study showed that senescence-induced oxidative stress may be an important contributor to link aging with endothelial dysfunction [39]. Ascites was also identified to be an important risk factor for VTE in OC patients receiving chemotherapy. Consistent with our systematic review, several studies showed that ascites was associated with VTE in patients with cancer, especially gynecological cancer [40–42]. Peripheral blood of patients with cancer is usually in hypercoagulable state, which may be worsened owing to ascites, a frequent event in patients with cancer, especially advanced cancer. Moreover, ascites was associated with reduced relative blood volume. All of these can explain the mechanism linking ascites to chemotherapy-associated VTE in OC. D-dimer level was regarded as a typical signature for VTE, and a meta-analysis also showed that the plasma D-dimer level correlated with disease progression and the VTE risk in patients with ovarian cancer [43]. In the current meta-analysis, only one included study found that D-dimer > 0.5 mg/mL was a significant risk factor for VTE in OC patients receiving chemotherapy. Interestingly, two included studies reported the role of chemotherapy-associated VTE on prognosis of OC patients with conflicting results [14, 27]. Regardless of the inconsistence, high level of plasma D-dimer level in OC patients undergoing chemotherapy should be vigilant and relevant preventive measures against VTE should be considered. BMI and obesity are reportedly associated with VTE in the general population [44–46], but the current available evidence in our systematic review did not determine whether BMI or obesity was a significant risk factor for chemotherapy-related VTE in OC. Fotopoulou et al. revealed that BMI < 30 kg/m^2^ was associated with approximately 3.2-fold increase of VTE in OC patients undergoing platinum/paclitaxel-containing first-line chemotherapy [27]. However, another study found that BMI < 30 kg/m^2^ was not a significant risk factor for chemotherapy-related VTE in OC [14]. Considering that hyperlipidemia was associated with VTE [47], the potential role of BMI or obesity in VTE development should be further studied. Therefore, whether BMI or obesity was a significant predictor for chemotherapy-related VTE in OC requires further investigation.

Several limitations also existed in our study. Firstly, the current study only included studies in Asia, Europe, and the USA, although we did not impose any restrictions in regions when we performed literature search. Therefore, regardless of the fact that the prevalence of chemotherapy-related VTE in OC was basically consistent in these regions, the results may not be generalizable to other non-involved areas, such as Africa and Australia. Secondly, there existed substantial statistical heterogeneity across included studies in the meta-analysis. Meta-regression analysis identified that sample size was a potential source of statistical heterogeneity. Irrespective of statistical heterogeneity, the results of subsequent subgroup analysis and sensitivity analysis were basically consistent with the overall pooled estimate, which indicated that the pooled prevalence was reliable and credible. Thirdly, the potential publication bias cannot be excluded in the meta-analysis, although we performed a comprehensive literature search. Regardless of the significant publication bias, the sensitivity analysis based on the trim-and-fill method indicated that the pooled estimate was basically consistent with the previous one after adding two “missing studies.” Finally, owing to inconsistent methodology description on risk factors in included studies, we only performed systematic reviews, but not meta-analysis for them, so correlative dimension of these risk factors with chemotherapy-related VTE was largely unclear. Moreover, there are other factors like anesthesia condition, clinic care, and other related health conditions which were not identified in the current meta-analysis, but may also be potential risk factors for chemotherapy-related VTE. Accordingly, these results may put cautious interpretation and further studies focused on these important risk factors should be warranted, which contributes to improving or controlling critical risk factors for chemotherapy-related VTE in OC.

## Conclusion

This current study revealed that the pooled prevalence of chemotherapy-related VTE in OC was approximately 9% in OC patients. Many risk factors were significant predictors for chemotherapy-related VTE including advanced age, D-dimer > 0.5 mg/mL, and tumor diameter > 10 cm. Future high-quality studies should be warranted to investigating the benefits of prevention strategies for chemotherapy-related VTE in OC patients.

## Supplementary Information

**Additional file 1.** The detailed search strategy.

## Data Availability

All the data and material are available.

## Abbreviations

OC: Ovarian cancer
VTE: Venous thromboembolism
PE: Pulmonary embolus
RR: Relative risk
OR: Odds ratio
CI: Confidence interval
NOS: Newcastle-Ottawa Scale
